# Supplementary material for: Polyketide synthase gene clusters in the ericoid mycorrhizal fungus Oidiodendron maius: genome-wide prediction, expression profiling, and functional characterization of knockout mutants
Source: Front Plant Sci. 2026 Sep 16;17:1924430. doi: 10.3389/fpls.2026.1924430 (PMC13623903; doi:10.3389/fpls.2026.1924430)
Supplement: Supplementary file 1 [file SupplementaryFile1.zip › Supplementary Materials/Supplementary Folder 1.zip!/Oidma1_AssemblyScaffolds_Repeatmasked/knownclusterblast/cluster58/jgi.p_Oidma1_36363_mibig_hits.html]

| MIBiG Protein | Description | MIBiG Cluster | MiBiG Product | % ID | % Coverage | BLAST Score | E-value |
| --- | --- | --- | --- | --- | --- | --- | --- |
| ADI24958.2 | GsfF | BGC0000070 | Griseofulvin\_biosynthetic\_gene\_cluster | 39 | 77.6923076923 | 79 | 3.2e-15 |
| BAI44340.1 | P450 | BGC0000677 | Cyclooctatin\_biosynthetic\_gene\_cluster | 38 | 56.1538461538 | 55 | 5e-08 |
| ADE22337.1 | monooxygenase | BGC0000065 | Galbonolides\_biosynthetic\_gene\_cluster | 32 | 56.9230769231 | 53 | 2.5e-07 |
| AEE83590.1 | cytochrome\_P450,\_family\_705,\_subfamily\_A,\_polypeptide\_4 | BGC0001313 | Arabidiol\_/\_Baruol\_biosynthetic\_gene\_cluster. | 30 | 66.9230769231 | 48 | 7.9e-06 |
